# Individual and Community-Contextual Level Factors Associated With Wellbeing Among Older Adults in Rural Zambia

**DOI:** 10.3389/ijph.2024.1606571

**Published:** 2024-02-19

**Authors:** Andrew Banda, Jaco Hoffman, Vera Roos

**Affiliations:** ^1^ Optentia Research Unit, North-West University, Vanderbijlpark, South Africa; ^2^ Department of Demography, Population Science, Monitoring and Evaluation, University of Zambia, Lusaka, Zambia; ^3^ The Oxford Institute of Population Ageing, University of Oxford, Oxford, United Kingdom

**Keywords:** individual and contextual factors, wellbeing, older adults, rural communities, Zambia

## Abstract

**Objective:** This article aims to identify individual and community-contextual level factors associated with the wellbeing of older adults (50 years and older) in rural Zambia.

**Methods:** Data from the nationally representative 2015 Living Conditions Monitoring Survey (LCMS) was used. Employing multilevel mixed effects, the individual and community-contextual factors on wellbeing were determined.

**Results:** Overall, 31.7% of rural older adults perceived their wellbeing as good. Both individual and community-contextual level factors are associated with the wellbeing of older adults in rural communities. At the individual level, wellbeing was associated with higher education attainment. Community-contextual factors significantly associated with wellbeing included improved housing, access to piped tap water within the premises, own charcoal or income to purchase firewood.

**Conclusion:** The findings foreground the imperative to analyse both individual and community-contextual level factors of wellbeing to generate and present evidence for investments in education across the life course and for the development of infrastructure towards increasing the wellbeing of rural older adults. Additionally, the results provide a basis for planning by devising policies and programmes for older people to thrive and for no one to be left behind regardless the setting.

## Introduction

As the global population ages, efforts to ensure older people’s wellbeing and quality of life, are becoming more prominent [1]. While rural areas worldwide face diverse and unique challenges providing social services due to resource constraints, geographical location, and diversity in cultural and social settings, the situation is more pronounced in developing countries [2, 3]. Developing countries will generally experience faster growth in absolute numbers of older people than developed countries [4]. For instance, the Sub-Saharan Africa (SSA) region is, population-wise, the youngest region [5], resulting in low prioritisation and implementation of ageing issues in national policies [6]. The region will experience the fastest growth rate in the absolute number of older people compared to any other region due to past fertility patterns and the current young age structure [7]. It is estimated to triple from 46 million in 2015 to 161 million by 2050 [8, 9]. Among SSA countries, Zambia has a young population with about 79% (15,570,950) under 35 years. The proportion of the population aged 50 years and over has steadily increased, averaging 8% (1,673,149) in 2022 and projected to grow to about 10% in 2035 [10, 11].

The 2022 Zambia Census of Population and Housing estimates that 6 out of 10 people live in rural areas [11], with most older people residing in rural areas where 79% of the general population is poor [12]. The rapid growth of the ageing population and the growing number of older people living in rural communities raise concerns about their socio-economic wellbeing, health and social care, the type of support available and access to daily living needs such as food, housing, energy and water to support their wellbeing [4, 13]. Limited infrastructure, economic constraints, changing social dynamics and cultural norms, coupled with persistent policy gaps, pose challenges for ageing well in rural communities [14, 15]. Rurality as such, and ageing processes associated with such settings make it contested spaces at the dynamic nexus of older people’s active and passive interactions with existing and potential community-contextual characteristics [14], impacting efforts towards the attainment of Sustainable Development Goals (SDGs), particularly those relating to health and social wellbeing.

The World Health Organisation (WHO) defines wellbeing as “a state of complete physical, mental, and social wellbeing, and not merely the absence of disease or infirmity” [16]. The WHO policy-oriented definition embodies aspects related to individual factors (e.g., health, education), and also community-contextual factors (e.g., access to services, general living conditions) [16, 17]; including the development and maintenance of positive interactions with local communities and contexts [18]. In its call for action to improve the wellbeing of older persons, The United Nations Decade of Healthy Ageing (2021–2030) positions communities as particularly important as they foster the abilities of older people by creating age-friendly environments that are good places to “grow, live, work, play, and age” [16]. We use community-contextual level factors to describe the tangible aspects of rural settings within which ageing and wellbeing are influenced.

There is growing interest in older people living in rural and remote areas as these locales face unique challenges and opportunities that affect their general wellbeing [2, 19]. Despite the often-perceived serenity of rural communities with strong social bonds and networks [20] as a distinctive feature, these areas generally have an older demographic profile with limited supportive services, often described as age-unfriendly resource-vulnerable settings [21].

Ageing in SSA rural communities particularly presents unprecedented socio-economic, cultural, structural, and public health challenges because of weak or non-existent policy frameworks on ageing [22]. Rural areas in Zambia, face disproportionately increased demands and associated costs in delivering health and social care services because of accessibility issues due to inadequate infrastructure and service limitations [23]. Rural communities tend to be geographically isolated due to a lack of investment in public transport and poor infrastructure to host and deliver essential services, in addition to low educational attainment among older adults [24] and high rural poverty [25]. The interplay of these factors in rural settings creates a challenging environment for older people’s wellbeing.

For ageing well in rural areas, Bosch-Farré et al. identify eight elements, namely: health, information, practical assistance, financial conditions, physical and mental activity, the company of friends and family, transport and safety [26]. Community-contextual characteristics for this article include environmental factors, accessibility of health and social services and the quality of available infrastructure [15]. The wellbeing of older Zambians also involves community support and care, anchored in the intergenerational extended family [27]. However, the family system is in flux [28, 29]: a dynamic compounded by the impact of HIV and AIDS, with a significant number of orphans left under the care of older people with no steady income to support themselves and their dependents [30, 31].

In response to rural ageing, a large body of literature has emerged on rural ageing, health systems, and economic and social implications in Europe and North America [32, 33], but much less about the factors of rural ageing and wellbeing in the least developed countries [34, 35]. The literature, therefore, broadly points to the inadequacy of community-context related factors in the analysis of wellbeing within rural settings. The gap identified, beckons scholars to move beyond a monolithic analysis of individual factors disaggregated by the blanket clustering of settings (broad rural or urban categorisations) towards an analysis of context-specific factors associated with the settings within which older persons live and through which they experience ageing. This dynamic interplay between older adults and relevant community-context characteristics requires further analysis to identify factors associated with older adults’ wellbeing. Such analysis considers complexity, here viewed through the lens of a critical realist approach that seeks to understand and explain complex relationships that underlie the social world and society’s perceived knowledge of it. Understanding the individual and community-contextual factors associated with the wellbeing of older people within the dynamic interplay with rural contexts provides an opportunity to promote older people’s wellbeing, thereby helping attain the goals of the 2030 Decade of Healthy Ageing [16], the Madrid Plan of Action [36], the AU Policy Framework and Plan of Action on Ageing (2022) as well as contributing to the rural ageing agenda as proposed by the Age-friendly cities/communities Framework [37].

This paper presents the individual, socio-economic conditions of rural older people and the rural community-contextual factors in understanding what influences the wellbeing of older people (50 years and older) in rural Zambia.

## Methods

### Data Source and Population

The data analysed in this study are from the 2015 LCMS, a nationally representative cross-sectional population-based household survey. The 2015 LCMS is the seventh wave in the series. Previous studies were conducted in 1996, 1998, 2002/2003, 2004, 2006, and 2010. The main aim of the LCMS is to monitor and highlight the living conditions of people. The LCMS collects information on the general living conditions, household income and expenditure, food security and coping strategies, economic activities, education attainment and health status of household members, housing conditions, as well as access to community-based facilities and services such as health facilities, banks and transport [24].

The 2015 LCMS covered 12,251 households in 664 randomly selected enumeration areas (EAs) across the ten provinces of Zambia. In the case of rural EAs, households were listed and stratified according to the scale of their agricultural activity areas (farming blocks as a way of demarcation typical for rural settings) [20]. Therefore, four explicit strata were created at the second sampling stage in each rural EA: the Small-Scale Agricultural Stratum (SSAS), the Medium-Scale Agricultural Stratum (MSAs), the Large-Scale Agricultural Stratum (LSAS) and the Non-Agricultural Stratum (NAS). In each stratum, 7, 5, and 3 households were selected from the SSAS, MSAS and NAS, respectively. In each rural EA, a minimum of 15 households were selected without large-scale agricultural households.

### Measures

**FIGURE 1:**
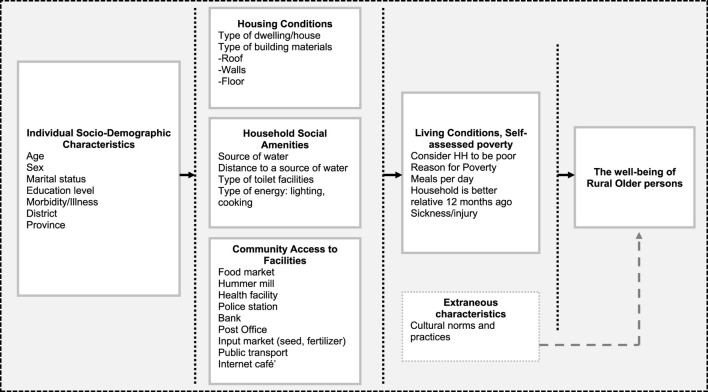
Factors influencing the wellbeing of rural older adults in Zambia (2015 Living Conditions Monitoring Survey, Zambia).

The outcome variable (wellbeing) was computed as a composite variable from four variables to assess access to amenities (facilities) in rural communities and self-assessed poverty—a three-response category measured self-assessed poverty: non-poor, moderately poor and poor. In assessing access to facilities, respondents were asked if they have a facility within the community, if they have used it in the last 12 months, and how far this resource is from the village. This analysis used these measures of self-assessed poverty and access to facilities because they provided a good indication of life satisfaction and the general living conditions of older people in rural Zambia. Figure 1 shows the summary classification of variables used in this study.

To assess wellbeing, a discrete binary variable coded as (1) if the respondent residing in the rural area described his/her household to be non-poor, has the facility within the community, has used the facility in the last 12 months, and the facility is within 5 km radius of the village (community); if otherwise, (0) is used.

The explanatory variables were categorised into two (2) broad categories: individual and community-contextual variables. Individual-level variables included the socioeconomic and demographic characteristics of older people, such as sex and age. The age of the respondents was categorised into intervals from 50–64, 65–74, and 75+. 75+ was coded in that manner because there were few older people in ages over 90 years. The level of education was categorised as 1 = primary education, 2 = secondary education, 3 = postsecondary education, and older people’s marital status was coded into three categories: 1 = single, 2 = married/living with a partner, 3 = divorced/separated and 4 = widowed. The general health wellbeing was assessed by whether an older person was ill or injured in the last 7 days before the survey and the number of meals per day.

Community-contextual variables included variables that described older people’s housing conditions and the type of material used for the walls, roofs, and floors. Housing variables were identified to provide the general living conditions or settings for older people. Four categorical variables were used: one variable described the type of dwelling (housing), and three variables were used to describe materials used for walls, roofs, and floors.

Similarly, access to water, type of toilet facility (sanitation) and the type of energy for cooking and lighting were used to describe further community-level elements that support older people’s wellbeing at the household level. Whether the house was connected to electricity was also included in the analysis. All these variables were categorical.

### Statistical Analysis

**FIGURE 2:**
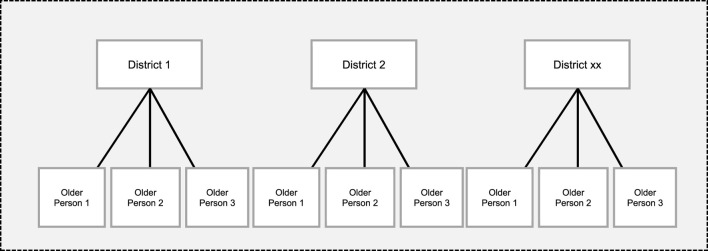
Hierarchical data structure, older people (N), level 1 are nested in districts (K), level 2 (2015 Living Conditions Monitoring Survey, Zambia).

The study analysis was performed in two steps. The first step involved descriptive and bivariate analysis in describing older people’s wellbeing by selecting explanatory characteristics (individual, household, and community characteristics). The second step involved multilevel regression modelling to measure the effect on the wellbeing of older people, first of individual characteristics: age, education attainment, morbidity (sickness); and second of community-contextual characteristics: type of dwelling, materials used for roof, walls and floor, source of water, and type of energy for cooking and lighting. Adjusted odds ratios (AOR) and a 95% confidence interval were used to report results. Multilevel regression was necessary because of the hierarchical nature of the data, which may violate one of the important assumptions of independence of the residuals [38] if ordinary logistic regression was used and may obscure factors of wellbeing that are a result of the hierarchical structure of older adults living in rural communities. Figure 2 shows the hierarchical data structure, in which older people (N) (the lower-level units) are nested in districts (K) (the higher-level units).

Figure 2 shows the data has a natural nested structure, where older people are nested in districts. The district was used as a unit of analysis because services are designed to cover the administrative level of the district. As such, all EA-level data were pulled into the districts they belong to.

A two-level multilevel analysis was used to examine the influence of individual and community-contextual factors on the wellbeing of older people. Older people (individual participants) constitute level 1. Older people are nested in districts which constitute level 2. In this analysis, districts are a level rather than a predictor/variable. On the other hand, variables such as education (no education, primary, and secondary level), marital status, type of housing, and water source are factors since their categories are both non-random and theoretically meaningful.

Multilevel regression analysis results were obtained using four (4) models. The null model (empty) was fitted without explanatory variables to predict random variability of the intercept and show the total variance in the wellbeing of older rural people. Model 1 examined the effects of individual-level characteristics of older adults on wellbeing. Model 2 examined the effects of community contextual-level characteristics, and Model 3 examined the combined effects of individual and community contextual-level characteristics, with results fixed at a 95% confidence level. The inter-class correlation (ICC) for each model was calculated to explain the proportion of variation attributable to the higher level of variation and compare models. The Proportional Change in Variance (PCV) was also calculated for each model regarding the empty model to show the power of the factors in the models in explaining the outcome variable.

Only significant variables from the bivariate and correlation analysis using Pearson’s chi-square test (*p* < 0.05) (5%) were added to the models. All analyses were conducted using Stata software version 14.0.

## Results

### Characteristics

A total of 14,531 older people’s data were captured for this analysis. In this case, 70 rural districts out of the total of 116 districts were included. The mean number of older people per rural district (*n* = 70) was 208, ranging from 34 to 663. Good wellbeing was experienced among 31.7% (95% CI: 30.739, 32.661) of older people (Table 2). Access to community facilities in rural areas was very low. Table 1 shows that only 15% and 12% of older people had used a facility and had a facility within a 5 km radius of the community (district), respectively.

**TABLE 1:** Summary description of contextual characteristics (*N* = 14,531) (2015 Living Conditions Monitoring Survey, Zambia).

Description	Count	%
The wellbeing of rural older adults	4,675	31.7
Access to Social amenities (facilities)		
Percent or rural adults reporting using a social amenity/facility(s)^a^	2,182	15.2
Percent of rural adults reporting having a facility (s)^a^ within 5 KM radius	1,660	12.1
Older People’s Self-Assessed Poverty Status		
Non-poor	1,196	15.2
Moderately poor	5,332	37.4
Very poor	7,996	55.6
Older People’s Self-Assessed Poverty Status relative 12 months ago		
Better off	2,669	18.2
The same	7,836	53.7
Worse off	4,009	28.1

^a^ Facilities included: Food market, Hummer mill, Health Facility, Police station, Bank, Post office, Farming Input market (Fertilizer, seed), public transport, internet café.

The average age of older people in the study was 62 (SD = 9.5), with the majority (63%) between 50–64 years. About 71% of older people were married or living with a partner, and 20% and 8% were widowed and divorced or separated, respectively. More than half of rural older people (58%) had a primary level of education, and 1 in 50 had a higher level of education. Among the total number of older people, the prevalence of morbidity in the last 7 days before the survey was 59%. There were significant relationships between wellbeing by gender (*p* < 0.01), level of education (*p* < 0.001), marital status (*p* < 0.001) and morbidity prevalence in the last 7 days before the survey (*p* < 0.05) (Table 2).

**TABLE 2:** Bivariate analysis of the wellbeing of rural older adults with individual and community-contextual characteristics in Zambia (2015 Living Conditions Monitoring Survey, Zambia).

Characteristics	Total *N* = 14,531	Wellbeing (older people 50+)		
		Yes (%)	95% CI	*p* -value
Age, m(SD)	61.7 (9.5)	61.8 (9.2)		
Age (Grouped)				
50–64	63.4	31.9	[30.8, 33.1]	*p* > 0.1
65–74	25.4	32.2	[30.3, 34.1]	
75+	11.2	296	[27.1, 32.3]	
Sex				
Male	72.6	32.4	[31.4, 33.5]	*p* < 0.01
Female	27.4	29.9	[28.2, 31.6]	
Level of Education				
No education	16.8	32.4	[30.2, 34.7]	
Primary	57.8	29.3	[28.1, 30.5]	*p* < 0.001
Secondary	23	34.3	[32.5, 36.2]	
Higher	2.3	62.5	[56.9, 67.8]	
Marital Status				
Never married	0.2	0.4	[0.2, 1.2]	
Married/Living with partner	71.1	31.9	[30.9, 33.0]	*p* < 0.001
Separated/Divorced	8	39.2	[35.8, 42.7]	
Widowed	20.7	28.4	[26.6, 30.4]	
Morbidity (Sick in the last 2 weeks)				
Yes (Sick, injured or both)	27.7	33.3	[31.5, 35.0]	*p* < 0.05
No	72.3	31.2	[30.1, 32.2]	
Number of Meals Per Day^a^				
One/two	58.7	28.6	[27.5, 29.7]	*p* < 0.001
Three or more	42.2	36.3	[34.8, 37.8]	
Province				
Central	14.8	30.7	[28.1, 33.4]	
Copperbelt	7.5	38.6	[36.2, 41.1]	
Eastern	17.4	34.3	[31.8, 36.8]	
Luapula	9.8	27.8	[25.3, 30.5]	
Lusaka	3.6	36.1	[32.9, 39.4]	
Muchinga	7.8	30.7	[28.1, 33.4]	
Northern	10.1	39	[35.9, 42.2]	*p* < 0.001
North Western	4.8	32.7	[28.8, 36.7]	
Southern	14.4	30.4	[27.9, 33.1]	
Western	9.9	20.7	[18.4, 23.3]	
Type of Dwelling (House)				
Traditional hut	49.8	27.1	[25.9, 28.4]	
Improved traditional house	31.9	30.6	[29.0, 32.2]	
Detached house	17.1	46.5	[44.1, 48.8]	*p* < 0.001
Flat/Apartment/multi-unit	0.3	24	[16.2, 33.9]	
Semi-detached house/servants’ quarter/cottage	0.9	53.4	[44.0, 62.5]	
Type of Materials Used for the Walls (House)^a^				
Mud brick	38.8	32.7	[31.2, 34.1]	
Burnt bricks	38.6	34.7	[33.2, 36.3]	
Compressed mud	10.2	28.8	[26.0, 31.8]	*p* < 0.001
Compressed cement bricks/concrete blocks/slab	0.6	60.8	[52.2, 68.9]	
Cement blocks	1.8	31.6	[25.8, 38.0]	
Iron sheets/asbestos/cardboard/wood/grass	1.3	15.9	[10.9, 22.6]	
Pole and dagga/mud	8.6	18.2	[15.8, 20.9]	
Type of Materials Used for Roof (House)^b^				
Thatched/palm leaf	55.4	28.2	[27.0, 29.4]	
Palm/Bamboo/wood planks/cardboard	0.6	45.9	[33.8, 58.4]	*p* < 0.001
Metal iron sheets	42.5	35.9	[34.5, 37.3]	
Asbestos	1.3	45.5	[38.3, 52.8]	
Type of Materials Used for Floor (House)				
Concrete	5.2	50.6	[46.4, 54.9]	
Cement	18.2	41.5	[39.2, 43.8]	
Brick	0.5	53.9	[38.7, 68.3]	
Tiles	0.2	31.4	[18.6, 47.7]	*p* < 0.001
Mud	75.1	28.1	[27.1, 29.1]	
Other	0.2	10.9	[5.6, 20.1]	
Don’t Know	0.6	16.4	[9.9, 25.8]	
Main Source of Water^c^				
Directly from river/lake/stream/dam/rainwater	17.9	30.3	[28.1, 32.6]	
Unprotected well	28	29.5	[27.9, 31.2]	
Protected well	12.8	35.5	[32.9, 38.2]	
Borehole	35.8	32.3	[30.7, 33.8]	*p* < 0.001
Protected spring	2.2	26.5	[21.7, 31.9]	
Public tap	1.4	47.4	[40.1, 54.8]	
Own tap	0.8	54	[46.1, 61.6]	
Other taps (nearby building)/Water Kiosk/Bought	0.9	37.7	[26.9, 49.8]	
Energy Used for Cooking^d^				
Collected firewood	87.5	30.6	[29.6, 31.6]	
Purchased firewood	1.5	60	[52.8, 66.7]	
Charcoal own product	3.7	35.1	[31.0, 39.5]	*p* < 0.001
Charcoal purchased	6.4	37.9	[34.7, 41.2]	
Electricity	0.9	43.6	[35.7, 51.9]	
Energy Used for Lighting				
Kerosine/paraffin/diesel	2.1	47.3	[40.8, 53.8]	
Electricity	1.5	56.5	[51.3, 61.6]	
Solar panel	7.5	43.6	[40.1, 47.2]	
Candle	7.4	35.2	[32.0, 38.5]	*p* < 0.001
Open fire	4.2	27	[23.1, 31.4]	
Torch	71.9	30	[28.9, 31.1]	
None	2.4	20.2	[15.7, 25.5]	
Other	2.9	26.3	[21.9, 31.1]	
Type of Toilet Facility^d^				
Own flush toilet inside/outside household	0.7	53.9	[46.5, 61.1]	
Own pit latrine with slab	11.4	41.5	[38.6, 44.5]	
Communal pit latrine with slab	2.4	24.3	[19.3, 30.1]	
Neighbours/another HH pit latrine with slab	0.5	55.3	[42.2, 67.7]	
Own pit latrine without a slab	53.1	31.2	[29.9, 32.4]	*p* < 0.001
Communal pit latrine without a slab	3.5	22.3	[18.2, 27.1]	
Pit latrine without a slab	18.8	30.4	[28.4, 32.5]	
None	4.4	27.6	[23.5, 32.1]	
Other	5.1	29.1	[25.1, 33.4]	

^a^ Missing.

^b^ 52 missing.

^c^ 39 missing.

^d^ 7 missing.

About half of older adults (49%) lived in traditional housing, with one in every five housing units (55%) used grass or leaves as materials for roofing (thatching) and about 4 in every 10 older adults in housing units (39%) constructed with mud bricks (Table 2). Concerning energy for cooking and lighting, only 2% of older adults in rural areas reported that their houses were connected to electricity, about nine in ten older adults (88%) collected firewood for cooking, and more than two-thirds (72%) used a hand-held torch for lighting. Regarding the type of toilet facilities, 53% were using a pit latrine (toilet) without a slab. About one-third (35%) of older adults accessed water from boreholes, 28% from unprotected wells and 18% from local water sources (e.g., rivers, lakes, streams, dams, rainwater (Table 3). There were significant differences in wellbeing in relation to: 1) the type of housing, 2) the type of materials used for the roofs, walls and floors, 3) the main source of water, and 4) the energy source for cooking and lighting (Table 2).

**TABLE 3:** Fixed and random effects result in the association of Wellbeing of rural older people with the individual and community-contextual factors in Zambia (2015 Living Conditions Monitoring Survey, Zambia).

Characteristics	Model 0		Model I		Model II		Model III	
	AOR	95% CI	AOR	95% CI	AOR	95% CI	AOR	95% CI
Age (Grouped)								
50–64			1	[1, 1]			1	[1, 1]
65–74			1.055	[0.97, 1.15]			1.08	[0.98, 1.19]
75+			1.923	[1.81, 1.05]			1.04	[1.07, 1.39]
Level of Education								
No education			1	[1, 1]			1	[1, 1]
Primary			0.945	[0.85, 1.06]			0.938	[0.84, 1.05]
Secondary			1.061	[0.94, 1.20]			0.931	[0.81, 1.06]
Higher			2.992***	[2.38, 3.77]			2.075***	[1.58, 2.73]
Morbidity (Sick in the last 7 days)								
Yes			1	[1, 1]			1	[1, 1]
No			0.944	[0.87, 1.03]			0.875**	[0.80, 0.96]
Province								
Central					1	[1, 1]	1	[1, 1]
Copperbelt					0.577	[0.25, 1.35]	0.564	[0.24, 1.34]
Eastern					0.765	[0.32, 1.84]	0.745	[0.31, 1.82]
Luapula					0.758	[0.31, 1.83]	0.759	[0.31, 1.85]
Lusaka					0.863	[0.29, 2.57]	0.88	[0.29, 2.67]
Muchinga					0.908	[0.37, 2.24]	0.874	[0.35, 2.18]
Northern					1.078	[0.46, 2.53]	1.068	[0.45, 2.55]
North-western					0.584	[0.25, 1.40]	0.573	[0.24, 1.38]
Southern					0.626	[0.27, 1.43]	0.609	[0.26, 1.41]
Western					0.839	[0.35, 2.04]	0.811	[0.33, 1.99]
Type of Dwelling (House)								
Traditional hut					1	[1, 1]	1	[1, 1]
Improved traditional house					1.273***	[1.11, 1.45]	1.281***	[1.12, 1.46]
Detached house					2.312***	[1.93, 2.76]	2.264***	[1.89, 2.71]
Flat/apartment/multi-unit					0.888	[0.50, 1.56]	1.055	[0.60, 1.86]
Semi-detached house/servants’ quarter/cottage					1.822**	[1.22, 2.71]	1.881**	[1.27, 2.79]
Type of Materials Used for Roof (House)								
Thatched/palm leaf					1	[1, 1]	1	[1, 1]
Palm/bamboo/wood planks/cardboard					1.003	[0.62, 1.63]	0.964	[0.59, 1.56]
Metal iron sheets					0.830*	[0.72, 0.96]	0.830**	[0.72, 0.96]
Asbestos					0.669*	[0.45, 0.99]	0.698^+^	[0.47, 1.04]
Type of Materials Used for the Walls (House)								
Mud brick					1	[1, 1]	1	[1, 1]
Burnt bricks					0.691***	[0.62, 0.78]	0.682***	[0.61, 0.77]
Compressed mud					0.859^+^	[0.72, 1.03]	0.862	[0.72, 1.03]
Compressed cement bricks/concrete blocks/slab					0.789	[0.49, 1.26]	0.759	[0.47, 1.23]
Cement blocks					0.116***	[0.07, 0.18]	0.098***	[0.06, 0.16]
Iron sheets/asbestos/cardboard/wood/grass					0.757	[0.48, 1.20]	0.784	[0.49, 1.24]
Pole and dagga/mud					0.757*	[0.61, 0.95]	0.780*	[0.62, 0.98]
Type of Materials Used for Floor (House)								
Concrete					1	[1, 1]	1	[1, 1]
Cement					0.612***	[0.51, 0.74]	0.582***	[0.48, 0.70]
Brick					1.428	[0.84, 2.44]	1.379	[0.80, 2.36]
Tiles					2.433*	[1.05, 5.63]	1.842	[0.75, 4.51]
Mud					0.590***	[0.49, 0.71]	0.561***	[0.46, 0.68]
Other					0.573	[0.27, 1.23]	0.533	[0.25, 1.15]
Don’t know					0.291***	[0.16, 0.54]	0.276***	[0.15, 0.51]
Main Source of Water								
Directly from river/lake/stream/dam/rainwater					1	[1, 1]	1	[1, 1]
Unprotected well					0.809**	[0.71, 0.92]	0.809**	[0.71, 0.92]
Protected well					1.073	[0.92, 1.26]	1.041	[0.89, 1.22]
Borehole					1.175*	[1.03, 1.34]	1.175*	[1.03, 1.34]
Unprotected spring					0.901	[0.70, 1.17]	0.899	[0.69, 1.17]
Public tap					2.521***	[1.70, 3.72]	2.493***	[1.68, 3.70]
Own tap					2.724***	[1.57, 4.72]	2.720***	[1.56, 4.76]
Other taps (nearby building)/water kiosk/bought					1.237	[0.80, 1.92]	1.303	[0.84, 2.02]
Energy Used for Cooking								
Collected firewood					1	[1, 1]	1	[1, 1]
Purchased firewood					3.625***	[2.75, 4.77]	3.349***	[2.54, 4.43]
Charcoal own product					1.359**	[1.12, 1.64]	1.376***	[1.14, 1.67]
Charcoal purchased					1.137	[0.97, 1.33]	1.074	[0.96, 1.26]
Electricity					0.425**	[0.24, 0.75]	0.281***	[0.15, 0.51]
Energy Used for Lighting								
Kerosine/paraffin/diesel					1	[1, 1]	1	[1, 1]
Electricity					0.827	[0.52, 1.34]	0.83	[0.51, 1.36]
Solar panel					0.535***	[0.40, 0.72]	0.527***	[0.39, 0.71]
Candle					0.564***	[0.42, 0.76]	0.572***	[0.43, 0.77]
Open fire					0.453***	[0.32, 0.63]	0.438***	[0.31, 0.62]
Torch					0.440***	[0.34, 0.57]	0.450***	[0.34, 0.59]
None					0.443***	[0.31, 0.64]	0.448***	[0.31, 0.65]
Other					0.333***	[0.24, 0.47]	0.317***	[0.22, 0.45]
Type of Toilet Facility								
Own flush toilet inside/outside household					1	[1, 1]	1	[1, 1]
Own pit latrine with slab					0.423**	[0.23, 0.78]	0.380**	[0.21, 0.71]
Communal pit latrine with slab					0.395**	[0.21, 0.75]	0.384**	[0.20, 0.74]
Neighbours/another HH pit latrine with slab					0.229***	[0.10, 0.50]	0.200***	[0.09, 0.44]
Own pit latrine without a slab					0.348***	[0.19, 0.64]	0.320***	[0.17, 0.59]
Communal pit latrine without a slab					0.167***	[0.09, 0.32]	0.153***	[0.08, 0.30]
Pit latrine without a slab					0.321***	[0.17, 0.60]	0.295***	[0.16, 0.56]
None					0.305***	[0.16, 0.59]	0.269***	[0.14, 0.52]
Other					0.348**	[0.18, 0.67]	0.316***	[0.16, 0.61]
Intercept	0.377***	[0.31, 0.46]	0.387***	[0.31, 0.49]	4.99***	[1.91, 13.04]	6.507***	[2.43, 17.41]
Random Effects								
Variance	0.658	[0.44, 0.98]	0.6502	[0.44, 0.97]	0.5504	[0.37, 0.83]	0.5657	[0.38, 0.85]
ICC (%)	16.7	[0.12, 0.23]	16.5	[0.12, 0.23/]	14.3	[0.10, 0.20]	14.7	[0.10, 0.21]
PCV (%)			1.2		16.4		14	
Model Statistics								
Log Likelihood	−8671.1		−8613.8		−8264.2		−8239.3	
AIC	17346.2		17243.7		16640.4		16602.5	
*N*	14,531		14,531		14,432		14,432	

Exponentiated coefficients; 95% confidence intervals in brackets ^+^ *p* < 0.1, *p < 0.05, **p < 0.01, ***p < 0.001.

Table 3 shows the multilevel mixed-effect results of individual and contextual factors associated with the wellbeing of older adults in rural areas. In the null model (Model 0), the wellbeing of older adults, the regional level variance was statistically significant with a variance level of 0.66 (*p* < 0.001). The ICC coefficients show that 17% of the variance in the wellbeing of older adults was attributed to differences in individual-level and community contextual-level factors. So, the inter-district differences were confirmed. The PCV in Model 1 shows that only 1% of the variation in the wellbeing of older adults was explained by individual-level factors. In Model 2, a PCV of 16% implies that variation in the wellbeing of older adults in rural areas was explained by community-level characteristics.

In Model 3, the results of a multilevel analysis on the wellbeing of older adults were statistically significant in relation to the individual-level variables (level of education and prevalence of morbidity). Concerning the contextual-level factors, the type of dwelling (house), materials used for roofs, walls, and floors, the main local water source, the type of energy used for cooking and lighting, and the type of sanitation service (toilet) statistically significant influenced older adults’ wellbeing in rural settings.

### Education Attainment and Morbidity

**FIGURE 3:**
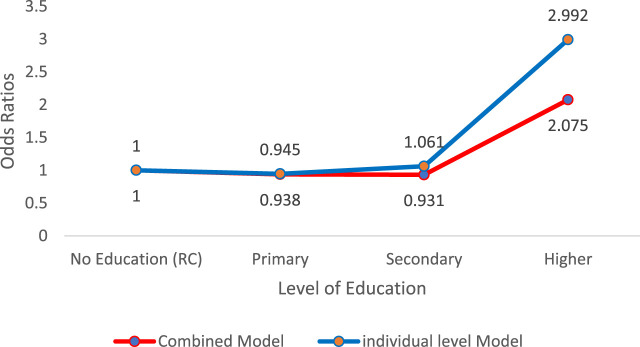
Distribution of odds ratios for older people’s wellbeing and level of education (2015 Living Conditions Monitoring Survey, Zambia).

The results show that older adults in rural areas with higher education attainment were more likely to experience good wellbeing compared to older adults with no education (AOR = 2.075, 95% CI: 0.58, 2.73) (Figure 3). The prevalence of morbidity (illness in the last 7 days) among rural older adults reduced the odds of wellbeing by 88% compared to older people who were not sick 7 days before the survey (AOR = 0.875, 95% CI: 0.80, 0.96).

### Housing

Housing conditions were an important element of wellbeing. Results showed that an improvement in the types of housing increased the wellbeing odds by 28% for older people who lived in improved traditional houses (AOR = 1.281, 95% CI: 1.12, 1.46) and doubled for those who lived in modern detached houses compared to older people who lived in traditional huts (AOR = 2.264, 95% CI: 1.89, 2.71).

### Water

Older adults with access to a borehole had 18% higher odds of wellbeing than older adults who accessed water directly from a river/stream/rainwater (AOR = 1.175, 95% CI: 1.03, 1.34). Similarly, older people who had access to a public tap (AOR = 2.493, 95% CI: 1.68, 3.70) and had their tap within the premises (AOR 2.720, 95% CI: 1.56, 4.76) as a source of water in rural areas were more than two times more likely to report good wellbeing than older adults who sourced water directly from rivers/lakes/streams/rainwater. The odds of wellbeing were generally lower for older people in rural areas without access to proper sanitation services (toilets).

### Energy

For older adults who purchased firewood as a source of energy for cooking, their odds of wellbeing were more than three times higher compared to older adults who collected firewood for this purpose (AOR = 3.349, 95% CI: 2.54, 4.43) and older people who had their charcoal had 38% higher odds of wellbeing compared to older adults who collected firewood (AOR = 1.376, 95% CI: 1.14, 1.67). Relatedly, among older adults whose source of energy for lighting was an open fire or other sources of energy, the likelihood of wellbeing decreased by 44% (AOR = 0.438, 95% CI: 0.31, 0.62) and 32% (AOR = 0.317, 95% CI: 0.22, 0.45), respectively.

The random effects in the final model show that the variance of the random intercept remained statistically significant across the models, suggesting divergence across the rural areas even after accounting for individual-level and contextual-level factors. This further suggests that other unmeasured or unobserved rural community characteristics may influence the wellbeing of older people. Although there are other unobserved rural community characteristics, the PCV of 14% indicates that the random effects (individual and contextual factors) included in the model account for the substantial portions of the variability in the wellbeing of older adults in rural communities. Therefore, unpacking the multilevel structure of the data is important to understand the context-specific nuances that influence the wellbeing of older adults in rural communities.

## Discussion

We aimed to identify individual and community-contextual level factors associated with the wellbeing of older adults above 50 years in rural settings. We established that both individual and community-contextual factors dynamically interact to influence rural settings that foster or hinder the wellbeing of older people. These findings align with other studies on rural ageing that suggest that rural settings are contested spaces for ageing and are created through active and passive interactions between diverse older adults, community members, rural organisations and the policy/programmatic architecture [14]. The study highlights that educational attainment at the nexus of access to adequate housing, the appropriate type of materials to construct housing, and access to water and energy for cooking create contested spaces. By identifying these spaces through the generation of evidence, possible opportunities are opened for policy and practical interventions that could be beneficial for ageing individuals and their communities.

At the individual level, higher education attainment among older adults was associated with better wellbeing. A population and development review argues that education attainment over the life course is a paramount driver for many social, economic and health outcomes [39]. Another study on the impact of education attainment on older people’s wellbeing found that each additional year of education attainment improved the wellbeing of older persons [40], also in terms of their social, economic and health outcomes [41]. Others have shown a qualitative increase in older people’s cognitive health, self-confidence, and life satisfaction with educational attainment [42]. The level of education has been argued to directly enhance the quality of social engagement and social interaction, which results in more opportunities for the formation of stronger social networks including connections with peers [43]—contributing to wellbeing.

Level of education correlates strongly with better job prospects, personal empowerment, and income. Better income in older age can reduce stress and contribute to general wellbeing. Importantly, education attainment can enhance health literacy and improve the ability to access, understand and use information to make informed health decisions for wellbeing. Although the results of this analysis have shown that the prevalence of illness or injury among older adults negates the gains in wellbeing, studies have indicated that older adults with higher levels of education have better health outcomes than their less-educated peers [41]. Education can facilitate and shape the wellbeing of older adults and is a key driver for attaining a demographic dividend and the SDGs. Education is a key mechanism to prepare for old age, especially when complemented by community-contextual factors such as social support, access to healthcare and a better socioeconomic status [27].

At the community-contextual level, the contested spaces for the wellbeing of older adults were associated with the available community resources, such as type housing, access to water, sanitation, and energy for cooking. The interactions with these (or lack of) resources, directly or indirectly shape the setting within which older adults age. Access to housing provides a sense of safety and increases the desire to age in a specific place [44]. The results have shown that older people with access to improved housing experienced better wellbeing than those who live in traditional huts. The results are consistent with the research reported in other studies, which argued that rural communities have distinctive challenges associated with infrastructure, specifically, and decent housing improves the general wellbeing of older people [15, 31].

The WHO further emphasises that housing protects people from hazards and promotes good health and wellbeing [36, 37]. However, another study in Zambia argues that the challenge related to housing dates to Zambia’s pre-independence times, and 80% of the national housing stock is in informal and unplanned settlements and made of poor materials not resistant to withstand an array of climatic and weather conditions [45]. The results also indicate that housing conditions in rural communities significantly impact individual older people and community wellbeing. Studies on health and housing have demonstrated that housing can affect various aspects of health, mental wellbeing, and overall quality of life [46].

According to the results, older adults living in improved houses with access to piped water and energy for cooking (such as sufficient income to buy charcoal) experienced better wellbeing than counterparts in poor housing with related conditions. The interaction of housing conditions, access to water and energy for cooking and lighting in rural settings directly influences older people’s wellbeing. Generally, most rural areas in Zambia face challenges concerning access to energy for cooking and lighting [24]. The results show that older people who purchased firewood or had charcoal for cooking had better wellbeing than older people in rural areas who collected firewood for cooking, given distances and weight. Access to water and sanitation services (toilets) remains a key wellbeing factor. Findings showed that older people in rural areas with access to either a borehole, public tap or a tap on their property have better wellbeing compared to older individuals who have to collect water directly from the source (e.g. river, lake, dam, rainwater). A possible explanation is that older people must walk long distances to the source of water, as a study by Koff confirmed, but also that many of them cannot carry heavy loads due to their frailty [15, 31, 47, 48].

In terms of a critical realist approach [49], it could be posited that the factors that support the wellbeing of older adults are obscured within the contextual causal relationship, as evidenced by the interaction of individual and rural community-contextual characteristics [50]. Thus, it is asserted that there is a need to move beyond a simplistic focus on older peoples’ observable individualistic characteristics towards a more complex understanding by integrating “real” world community-contextual effects, evidenced in this study. The monolithic clustering and characterisation of older people based on the binary/blanket categorisation of communities as rural and/or urban are likely to obscure a true reflection of the wellbeing of older adults. Consequently, the analysis of older adults’ wellbeing should consider the specific characteristics of individuals at the interface of the particular rural context.

The findings suggest that the community-contextual factors of wellbeing are diverse and dynamic. As such, the emergence of any external influence could threaten elements that support contested spaces beneficial for the wellbeing of older adults. For example, the COVID-19 pandemic of 2020 negatively affected the elements that create a favourable setting for the wellbeing of older people, such as loss of income, inadequate food, challenges to access healthcare, and exacerbated isolation due to restricted movements [51, 52]. The 2021 Socio-economic Impact Assessment Survey of COVID-19 on Households in Zambia (SEIA) highlights how COVID-19 altered mechanisms for the wellbeing of older adults. Thus, any efforts that do not consider the variability of community-contextual characteristics in understanding what influences the wellbeing of older adults may not generate optimal outcomes.

The stark reality is that only about a third of rural older adults in this study experienced wellbeing. It is therefore imperative to highlight the identified factors that facilitate wellbeing with a clear and critical realist approach to structure attainable interventions that may otherwise be obscured in the reductionism and clustering of the challenges in rural communities. This implies that the factors that support the wellbeing of older people in rural communities should be looked at with a three-tier approach by focusing on what is *prevailing* in rural communities, the underpinning factors i*nfluencing* the prevailing factors, and how they *interface* with the prevailing wellbeing of older adults.

### Conclusion

This study adds compelling evidence to the studies about rural ageing in SSA on the influence of individual factors (education attainment) and community-contextual factors (access to improved housing, piped water, having own energy sources for cooking such as charcoal or income to buy firewood) on the wellbeing of older people in rural communities. These results underscore the need to address educational disparities and improve access to basic community resources to promote the wellbeing of older populations in rural communities. Furthermore, this analysis has policy-making and pragmatic implications. To this end, the 2022 African Union Strategic Policy Framework and Plan of Action on Ageing (AUPFPAA) calls for strategic investment across the life course (in this case, education) to enhance capacities and wellbeing in older age that can benefit both older and younger people [53]. This might, in turn, foster the attainment of a demographic dividend as the population ages. These results also inform a call for direct investment in rural infrastructure such as housing, water access, and energy (cooking, lighting). Amalgamated efforts are needed to negotiate and address the contested spaces for rural ageing by valuing the participation and needs of current cohorts of older citizens and, to that end, also investing in future generations through education. It emphasises the call for a life course approach to wellbeing in later life through education, as well as the need to ensure that older people’s physical environments are good or friendly places to age.

The limitations in terms of the dataset are acknowledged on two levels: the use of the 2015 data may have presented some inadequacies due to it being dated and potential changes might have occurred, resulting in changes in the context; the data were also collected to measure the general wellbeing of the population. This focus may have missed salient aspects unique to older adults. Nevertheless, the results point to a non-monolithic analysis of what shapes the wellbeing of older adults by interfacing individual and community-contextual level factors. The multi-level analysis has demonstrated the need to decrypt factors of wellbeing often obscured in the monolithic analysis of individual or community-contextual level factors separately. This is because the monolithic approach might risk not recognising the diverse, dynamic and complex interface of individual and community-contextual factors for the wellbeing of older adults. Further research is required to explore additional determinants of wellbeing, specifically human and social capital, and the development and impact of specific community-context interventions to support the wellbeing of older people in rural settings.

## Data Availability

Data are available at the Zambia Statistics Agency (https://www.zamstats.gov.zm) for public use upon a data request through the Statistician-General.
